# Comparison of nutritional value of the wild and cultivated spiny loaches at three growth stages

**DOI:** 10.1515/biol-2022-0969

**Published:** 2024-10-25

**Authors:** Zeguo Zeng, Qingxiang Zeng, Xinmin Lu, Miao Zheng, Yuan Fang, Jing Guo, Fang Luo, Xiaorong Zeng, Zhihuan Cai, Bin Liu, Lifang Deng, Fei Zeng, Xianguo Zou

**Affiliations:** Ganzhou Animal Husbandry and Fisheries Research Institute, Gannan Academy of Sciences, Ganzhou, 341000, People’s Republic of China; Agricultural Technology Promotion Center of Deqing County, Deqing 313200, Huzhou, People’s Republic of China; Agricultural Technology Promotion Center of Ganzhou, Ganzhou, 341000, People’s Republic of China; College of Food Science and Technology, Zhejiang University of Technology, Hangzhou 310000, People’s Republic of China; Agriculture and Rural Bureau of Ruijin, Ruijing 342500, People’s Republic of China; Agricultural Technology Promotion Center of Yudu, Yudu 342300, Ganzhou, People’s Republic of China

**Keywords:** spiny loach, proximate compositions, fatty acids, amino acids, nutritional value, farming

## Abstract

Environmental pollution and overfishing of wild spiny loach have led to the increased demand for breeding the fish. However, the nutritional value between the wild and cultivated spiny loaches was unknown. Therefore, this study aimed to evaluate the nutritional components among the wild and cultivated spiny loaches at different growth stages by analyzing and comparing the proximate compositions, fatty acids, amino acids and volatile compounds. Results showed that the cultivated ones had significantly higher energy and fat contents than the wild. Particularly, the cultivated second-age spiny loach contained the highest contents of polyunsaturated fatty acids (4.83 ± 0.01%) and EPA + DHA (0.85 ± 0.02%). Besides, the total essential amino acid content of cultivated second-age spiny loach was 2201.28, exceeding that recommended in the FAO/WTO scoring pattern (2,190). And it had the highest flavor amino acid (6.24 ± 0.04 g/100 g), essential amino acid index value (71.82) and higher contents of volatile compounds. Overall, the cultivated spiny loach, especially that at the second growth stage, displayed the highest nutritional value. The findings of this study would help farmers to harvest the suitable breeding stage of spiny loaches from the perspective of nutritional value, which is beneficial to the sustainable fish farming.

## Graphical abstract

**Figure:**
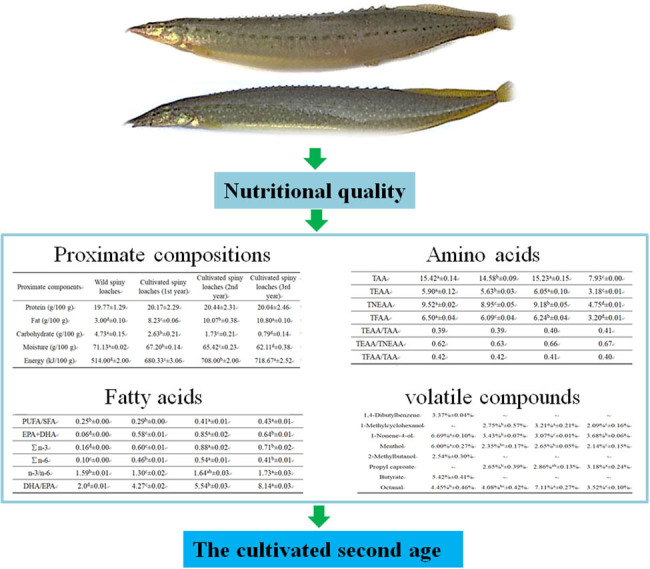

## 1 Introduction

The spiny loach (*Mastacembelus armatus*), having the characteristics of a row of separate spines on the back, is mainly distributed in the South Asian subcontinent and Southeast Asia, and the wild feeds on aquatic insects and small fish [1]. Spiny loach is considered an integral part of a healthy diet as it is abundant in nutritional components, such as amino acids, fatty acids and volatile compounds [2]. Amino acids are precursors of many flavors that can indirectly affect the taste, particularly for flavor and odor compounds [3], and play key roles in intestinal metabolism, cell signaling, gene expression, immune and anti-oxidative responses [4]. Fat of loach was reported to contain rich long-chain *n*-3 polyunsaturated fatty acids (LC *n*-3 PUFA), especially eicosapentaenoic acid (EPA) and docosahexaenoic acid (DHA). Indian spiny loach was detected to have 16.59% LC *n*-3 PUFA and 15.5% EPA + DHA expressed in total area [5]. The LC *n*-3 PUFA was very useful for reducing obesity, including suppression of appetite, enhancement of fat oxidation and energy expenditure and reduction of fat deposition [6]. EPA and DHA can help improve blood circulation and promote brain development, thus has the benefit to improve cardiovascular health and reduce cardiovascular disease (CVD) risk [7]. Dietary foods rich in LC *n*-3 PUFA such as fish and vegetable oils were reported to have anti-inflammatory properties, reduce insulin resistance and protect against metabolic syndrome [8,9]. Volatile compounds, such as (E)-2-hexenal, hexanal, linalol and a-terpineol, exerted antioxidant, anti-inflammatory, anti-cancer and anti-obesity activities [10].

Growing world population and increased awareness of the healthy benefits of aquatic products have consistently increased global demand of spiny loach [11]. In recent years, due to overfishing and environmental pollution as well as the slow growing speed, the number of wild spiny loaches has declined sharply, and thus cannot satisfy market demand. Some scholars have investigated the current situation of wild spiny loach resource in Taojiang river (Hunan province, China), showing that the output in traditional fishing sites is declining year by year, and the annual production of the spiny loaches in 2018 was only 10% of the historical maximum. Similar situations also occurred worldwide. Therefore, the aquaculture industry of spiny loach is a suitable and sustainable choice to increase the global supply of loach [12,13].

With the rapid upgrading of consumption, fish quality is an important concern for consumers [14]. Relevant studies have shown that farmed aquatic foods have an advantage over captured fishery products because they are produced and harvested under controlled conditions, which allow consumption-related risks to be minimized. At present, most of the spiny loaches in the market are cultivated, but there are few studies regarding comparing the nutritional value of cultivated and wild spiny loaches. So the question of who is healthier, either wild or cultivated loaches, needs to be illustrated.

Although scholars have carried out some studies on the nutritional components in the muscle of loach and the fatty acid compositions of males’ and females’ loach in the reproductive season [15,16], there is still no research on the nutritional components of the cultivated loach at different growth stages, especially fatty acid compositions. Therefore, it is necessary to carry out the nutrient detection and analysis of the spiny loach between the wild and cultivated. In this study, the nutritional components of the wild and cultivated spiny loaches at three different ages, including protein, fat, fatty acids, amino acids and volatile compounds were detected and compared. Results will help farmers choose cultivated spiny loach of suitable stage to improve its quality and thus satisfy consumers’ nutritional need.

## 2 Materials and methods

### 2.1 Sampling and sample preparation

**Figure 1:**
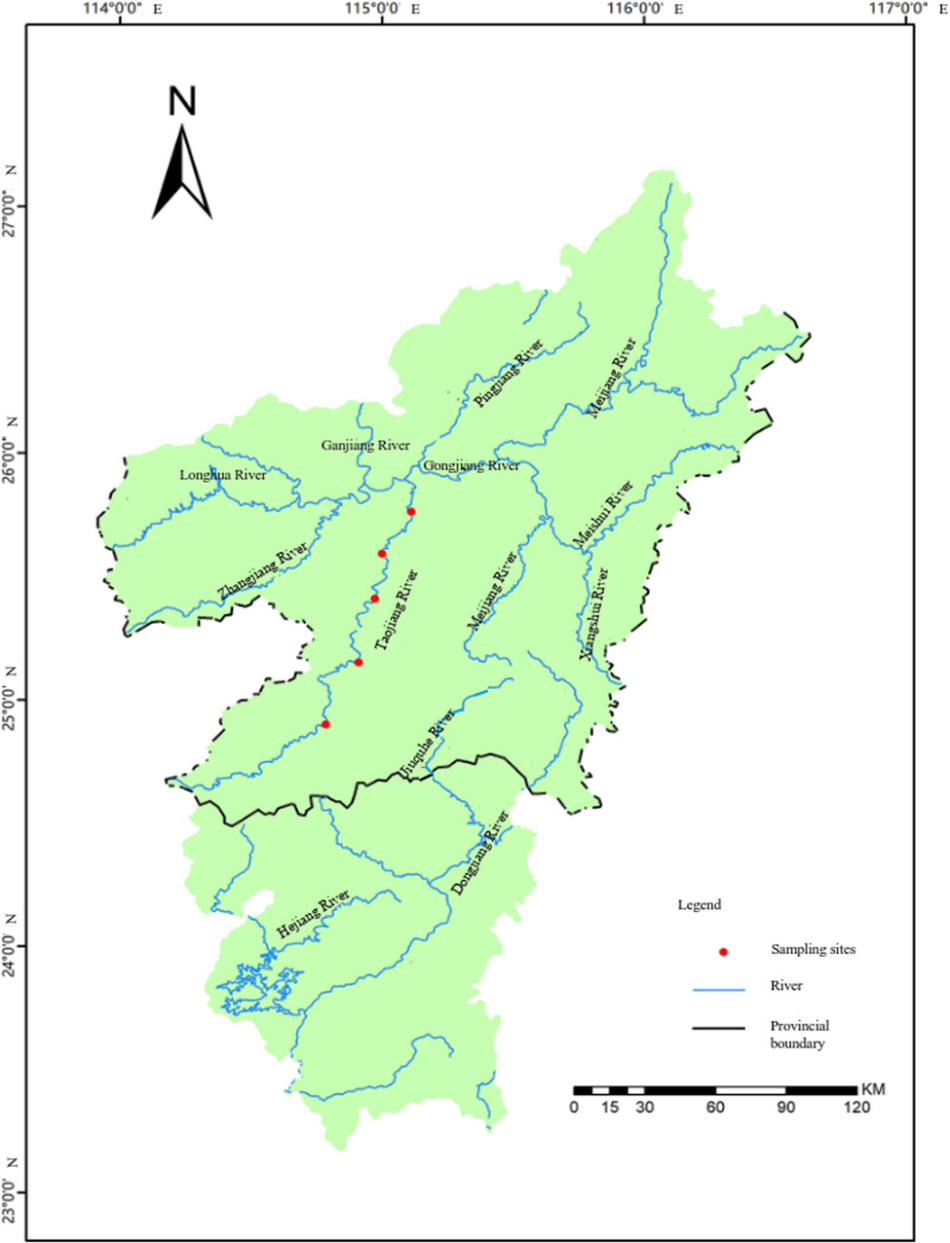
The sampling sites of wild spiny loaches in Taojiang River.

The wild specimens of spiny loaches were collected from Taojiang River (Hunan, China) using fishing cage in August 2020 (Figure 1). The wild spiny loaches live in ecological breeding condition with water depths of 2 m and water temperatures of 10–30ºC. After identification by professionals, 20 wild spiny loaches at the second age were used for the experiment. The farmed spiny loaches were from the first offspring of wild parents and were cultivated in Ganzhou Animal Husbandry and Fisheries Research Institute, feeding with the formula fodder (crude protein ≥ 43%, crude fat ≥ 15%, carbohydrate ≤ 4%, crude ash ≤ 18%, lysine ≥ 2.1%, total phosphorus ≥ 1.0%, calcium 2.0–5.0%), under the conditions of imitation ecological breeding. The cultivated specimens of 20 spiny loaches at the first, second and third age were collected in August 2020, 2021 and 2022, respectively.

After cleaning, dissecting and skinning, muscles from the back of the head to the front of the caudal stalk in the wild or the cultivated specimens were quickly taken, and were homogenized in a mixer for 1 min at 1,500 rpm prior to analysis, with size appropriate for individual analytical tests (10–100 g). All the subsamples of the homogenate were stored in sterile polypropylene containers in a deep freezer at −80°C until analysis was performed.


**Ethical approval:** The use of wild fish was officially approved by the Department of Agriculture and Rural Affairs of Jiangxi Province (Nongbanchangyu 2021-2). The research related to animals’ use has been complied with all the relevant national regulations and institutional policies for the care and use of animals.

### 2.2 Determination of proximate compositions

The chemical compositions (moisture, crude fat and crude protein) of all the samples were determined according to the Association of Official Analytical Chemists (AOAC) official procedures [17]. For the detection of moisture (AOAC 950.46), samples were dried in oven at 103°C for 8 h. Crude fat (AOAC 948.15) was determined by gravimetric method after the Soxhlet extraction, in which the samples were digested with acid hydrolysis and the fats were then extracted with petroleum ether using a Soxtec 2050 automated device (Foss, Shanghai, China). Crude protein (N × 6.25) (%) was detected by the Kjeldahl method (AOAC 981.10) using a Digestion Unit 8-Basic and an NKD6260 automated distillation and titration device (Foss, Shanghai, China).

The energy values were calculated using the mean values of protein and lipids in the spiny loach with the reference to the method reported by Usydus et al. [18]. The calculations were made with the following energy equivalents.

− protein: 17 kJ/g
− lipids: 37 kJ/g.

### 2.3 Determination of fatty acids by GC-FID

Fatty acid contents were detected according to our previously published methods [19]. Approximately 25  ±  0.1 mg of lipid sample were inserted into a tube, and added with 500 µL of methyl tricosanoate (1 mg/mL) and 4 mL of a 0.5 mol/L NaOH solution in methanol. Then the tube was closed and placed in an ultrasonic bath at room temperature (25°C) for 5 min. After that, 5 mL of esterifying reagent was added, and the tube was once again closed and placed in the bath for 6 min. Then, the tube was definitely removed from the bath and 4 mL of a saturated sodium chloride solution was added, and the entire system was closed and vigorously stirred for 30 s. Approximately 2 mL of *n*-hexane was added and then the tube was closed again and stirred for 30 s. After 24 h of rest under −18°C, the organic phase of tube was collected for chromatographic analysis. The chromatographic separation was performed using a molten quartz capillary column (100 m × 0.25 mm × 0.2 μm, CP-Sil 88, Chrompack; Agilent, USA); the carrier gas was H_2_, and the combustion gas was N_2_, H_2_ and air. The temperature procedure was 45°C for 4 min, increased up to 175°C at a rate of 13°C/min and maintained for 27 min, then further increased to 215°C at a rate of 4°C/min and held for 35 min, and the total running time was 86 min. The hydrogen flow rate was 30.0 mL/min, the air flow rate was 300 mL/min, and the nitrogen flow rate was 30.0 mL/min. Fatty acid methyl esters (FAMEs) in fish samples were identified by comparing their retention times with those from the standard FAME mixture, and quantified using tridecanoic acid methyl ester as internal standard. For the content of fatty acids in samples, the data were expressed in g/100 g of fresh weight.

### 2.4 Determination of volatile compounds by GC-MS

The volatile compounds of spiny loach were detected via headspace solid-phase micro-extraction combined with gas chromatography-mass spectrometry (GC-MS) according to the method of Rao et al. [20] with minor modification. One gram of fresh spiny coach was homogenized and transferred to a 20 mL sealed extraction bottle. Volatiles were extracted using a 50/30 μm DVB/CAR/PDMS coated fiber (Zhenzheng Analytical Instrument Co., Qingdao, China) at room temperature for 30 min. The fiber was then inserted into the sample injector of a GC-MS instrument (Trace1300-ISQLT, Thermo Fisher, USA) and desorbed for 5 min at 250°C. The working conditions of GC-MS were as follows: Thermo Trance TG-5MS GC column (30 m × 0.25 mm × 0.25 μm); temperature procedure: 40°C for 2 min, increased to 130°C at a rate of 5°C/min and maintained for 1 min, then further increased to 280°C at a rate of 15°C/min and maintained for 2 min. Mass spectra were detected at the *m*/*z* range of 35–500 with an electronic impact energy of 70 eV and a quadrupole temperature of 250°C. The volatile compounds of spiny loach were identified by comparing with that in the NIST MS 2.2 library at a criterion of at least 75% similarity. The concentrations of volatile compounds in samples were calculated as the percentage of individual peak areas relative to the total peak area.

### 2.5 Determination of amino acids by HPLC [21]

Spiny loaches were homogenized and dried at 105°C, ground into powder and passed through 40 mesh sieve. One gram of spiny loaches were added with 6 mol/L HCl, and then hydrolyzed at 110°C for 24 h. The hydrolysates were concentrated and dried by evaporation. Then the dried samples were dissolved in 0.02 M HCl (6 mL) and passed through a 0.22 μm filter membrane (Merck, Darmstadt, Germany) to remove impurities. An aliquot of 20 μL of filtrate was added to the automatic Model L-8900 Amino Acid Auto-Analyzer (L-8900; Hitachi, Japan) with analytical C18 column (4.6 × 150 mm, 5 μm; Agilent Technologies), and each amino acid was identified with the reference to amino acid standards and quantified by an internal standard phenyl isothiocyanate. For the content of amino acids in samples, the data were expressed in g/100 g of fresh weight. Total amino acids (TAAs) were calculated as the sum of each amino acid.

### 2.6 Calculation of amino acid score (AAS), chemical score (CS) and essential amino acid index (EAAI)

AAS and CS were calculated according to the equations of FAO/WHO [22], also reported by Oztekin et al. [21].

\[\text{AAS}\hspace{.5em}\text{=}\hspace{.5em}{\text{AA}}_{\text{FBP}}/{\text{AA}}_{\text{FW}},]\]

where AA_FBP_ is the concentration level of amino acid per test protein (mg/g, FBP: fish body protein) and calculated as follows:

\[{\text{AA}}_{\text{FBP}}\hspace{.5em}\text{=}\hspace{.5em}(\text{essential amino acid/crude protein})\left\times\text{6.25}\left\times\text{1,000,}]\]

and AA_FW_ shows the level of amino acid per protein with the reference to composition of FAO/WHO standard (mg/g) as given in Table 4.

\[\text{Chemical}\hspace{.5em}\text{Score, CS = }{\text{(AA}}_{\text{FBP}}\text{)}/{\text{(AA}}_{\text{EGG}}\text{)},]\]

where AA_EGG_ represents the concentration of amino acid per protein referred to the composition (mg/g) of whole egg protein (mg/g) as listed in Table 4.

The EAAI was calculated according to Oztekin et al. [21]:

\[{\mathrm{EAAI}}=\sqrt[n]{\left[(100\times {{\mathrm{EAA}}}_{1}\left/{{\mathrm{EAA}}}_{1{\mathrm{EG}}})\left\times (100\times {{\mathrm{EAA}}}_{2}\left/{{\mathrm{EAA}}}_{2{\mathrm{EG}}})\times ...\hspace{.2em}\left\times \hspace{.3em}(100\times {{\mathrm{EAA}}}_{n}\left/{{\mathrm{EAA}}}_{n{\mathrm{EG}}}\left)]},]\]

where “*n*” is the number of amino acids (considering pairs such as methionine + tyrosine).

EAA_1_, EAA_2_, … EAA_*n*_ are the levels of EAAs per test protein. EAA_1EG_, EAA_2EG_, …, EAA_*n*EG_ are the levels of EAAs per test protein of the egg reference concentration.

### 2.7 Statistical analysis

Statistical analyses in this study (proximate composition, fatty acid compositions, amino acid compositions and volatile compounds) were conducted in triplicate to minimize deviation, and data were presented as mean ± SD. The values for above analyses among the fish species were subjected to homogeneity and normality tests. When the assumptions were met, one-way ANOVA and Turkey HSD test with the help of the SPSS 17.0 software were used to determine the statistical significance. A value of *p* < 0.05 indicated statistical significance.

## 3 Results and discussion

### 3.1 Proximate composition

The basic nutrients of the wild and cultivated spiny loaches in three cultivation stages are shown in Table 1. Protein contents of spiny loaches in four groups were 19.77, 20.17, 20.04 and 20.44 g/100 g, respectively, showing no significant differences.

**Table 1:** Proximate composition of the wild and cultivated spiny loaches at three growth stages

Proximate components	Wild spiny loaches	Cultivated spiny loaches (1st year)	Cultivated spiny loaches (2nd year)	Cultivated spiny loaches (3rd year)
Protein (g/100 g)	19.77^a^ ± 1.29	20.17^a^ ± 2.29	20.44^a^ ± 2.31	20.04^a^ ± 2.46
Fat (g/100 g)	3.00^d^ ± 0.10	8.23^c^ ± 0.06	10.07^b^ ± 0.38	10.80^a^ ± 0.10
Carbohydrate (g/100 g)	4.73^a^ ± 0.15	2.63^b^ ± 0.21	1.73^c^ ± 0.21	0.79^d^ ± 0.14
Moisture (g/100 g)	71.13^a^ ± 0.02	67.20^b^ ± 0.14	65.42^c^ ± 0.23	62.11^d^ ± 0.38
Energy (kJ/100 g)	514.00^d^ ± 2.00	680.33^c^ ± 3.06	708.00^b^ ± 2.00	718.67^a^ ± 2.52

Note: The different letters (a, b, c and d) in the same line indicated significant differences (*p* < 0.05).

The fat content of wild spiny loaches was 3.00 g/100 g, significantly lower than those of cultivated spiny loaches at three growth stages (8.23, 10.07 and 10.80 g/100 g), suggesting that cultivated spiny loaches are medium fatty fish with fat content greater than 5% by weight [23]. Similarly, the energy in the wild loaches was also the lowest (514 kJ/100 g) compared with the cultivated ones (680.33–718.67 kJ/100 g).

Water content of the wild spiny loaches was the highest, and with the growth of age, the water content of the cultivated loaches gradually decreases, floating at the range of 71.13–64.11 g/100 g. The wild spiny loach group had the highest carbohydrate content of 4.73 g/100 g, while the carbohydrate content of cultivated spiny loaches decreased with the increased age at 2.63, 1.73 and 0.79 g/100 g, respectively. This might have resulted from their fodder, as the wild spiny loaches feeding on aquatic insects and small fish, the cultivated ones feeding on formula fodder with carbohydrate below 4%.

### 3.2 Fatty acid compositions

Fatty acid content is an important indicator to evaluate the nutritional value of fish. It is well established that saturated fatty acids (SFAs) increase low-density lipoprotein cholesterol, a strong risk factor for CVD [24]. When the intake of unsaturated fatty acids is insufficient, it will cause CVD and cerebrovascular disease and tumors, and the loss of omega-3 fatty acids (especially EPA and DHA) will cause the lack of nutrients in the brain, thus affecting thinking and memory [25]. The fatty acid contents of four groups are shown in Table 2; the SFA, MUFA and PUFA were 1.06–3.60, 0.97–4.83 and 0.26–1.42 g/100 g fresh weight, respectively. The SFA of cultivated spiny loaches was significantly higher than that of the wild (1.06 ± 0.00 g/100 g fresh weight), and spiny loaches at the first- and second-age groups were found to have the highest SFA contents (3.60 ± 0.03 and 3.45 ± 0.01 g/100 g fresh weight). MUFA accounted for the highest content among all fatty acids, and the data were similar to that reported from fish of sea bass and sea bream [26]. The MUFA of cultivated spiny loaches was significantly higher than that of the wild (0.97 ± 0.01 g/100 g fresh weight), and the highest MUFA content was found in the second-age group (4.83 ± 0.01 g/100 g fresh weight). PUFA in four groups showed similar trend with MUFA, and the highest PUFA content was still found in the second-age group. PUFA/SFA ratio is an important value to access the lipids, and the value above 0.4–0.5 is required if a diet is to combat various lifestyle diseases. The recommended PUFA/SFA ratio was only met by the cultivated spiny loaches at the second/third age, having the highest PUFA/SFA ratio (0.41 or 0.43). Numerous studies reported that dietary foods rich in MUFA and PUFA exhibited protective role against cardiovascular events, non-alcoholic fatty liver, inflammation and oxidative stress [27,28]. Palmitic acid (C16:0), oleic acid (OA, C18:1), palmitoleic acid (C16:1) and DHA were found in high levels. The content of C18:1 (OA) in the wild spiny loach was 0.58 ± 0.02 g/100 g fresh weight, significantly lower than the cultivated ones, 2.95 ± 0.01, 3.49 ± 0.03 and 2.56 ± 0.02 g/100 g fresh weight, respectively. This might be due to the higher fat content (≥15%) in the artificial feed. It has been reported that the amount of C18 fatty acids (such as OA, linoleic acid and α-linolenic acid) in farmed fish increased after using vegetable oil in feed [29]. This class of fatty acids is considered to have high nutritional value because they protect against CVD and contribute to the enrichment of aromatic components [30,31,32]. The current study indicated that the *n*-3:*n*-6 ratios in the wild and cultivated spiny loaches at the second/third age were much higher than that in the cultivated at the first age. In addition to the food they consumed, this ratio depends largely on their breeding stage. Fatty acids with good *n*-3:*n*-6 ratio (1.30–1.73) can potentially reduce the risk of CVD, neural disorders and cystic fibrosis [33]. Supporting our results, Manoharan et al. [5] found that fish *Lepidocephalus thermalis* had higher *n*-3:*n*-6 ratio of 1.19–1.97. Özogul et al. [34] reported 1.7 for European seabass caught off from the coast of Turkey.

**Table 2:** Fatty acid contents between the wild and cultivated spiny loaches (g/100 g fresh weight)

Fatty acids	Wild spiny loaches	Cultivated spiny loaches (1st year)	Cultivated spiny loaches (2nd year)	Cultivated spiny loaches (3rd year)
C12: 0^*^	0.01 ± 0.00	—	—	—
C14: 0^*^	0.09^d^ ± 0.00	0.33^a^ ± 0.00	0.31^b^ ± 0.00	0.22^c^ ± 0.00
C15: 0^*^	0.02^a^ ± 0.00	0.01^b^ ± 0.00	0.01^b^ ± 0.00	0.01^b^ ± 0.00
C16: 0^*^	0.71^d^ ± 0.00	2.86^a^ ± 0.03	2.63^b^ ± 0.01	1.98^c^ ± 0.03
C17: 0^*^	0.03 ± 0.00	—	—	—
C18: 0^*^	0.16^d^ ± 0.00	0.39^b^ ± 0.00	0.49^a^ ± 0.01	0.35^c^ ± 0.01
C21: 0^*^	0.01 ± 0.00	—	—	—
C24: 0^*^	0.03^a^ ± 0.00	—	0.01^b^ ± 0.00	0.01^b^ ± 0.00
C14: 1 *n*-5^#^	0.02^a^ ± 0.00	0.01^b^ ± 0.00	0.01^b^ ± 0.00	0.01^b^ ± 0.00
C16: 1 *n*-7^#^	0.32^c^ ± 0.01	1.10^a^ ± 0.02	1.13^a^ ± 0.02	0.87^b^ ± 0.03
C17: 1 *n*-7^#^	0.02^a^ ± 0.00	—	0.01^b^ ± 0.00	—
C18: 1 *n*-9 (OA)^#^	0.58^d^ ± 0.02	2.95^b^ ± 0.01	3.49^a^ ± 0.03	2.56^c^ ± 0.02
C20: 1 *n*-9^#^	0.02^d^ ± 0.00	0.15^c^ ± 0.00	0.18^a^ ± 0.00	0.16^b^ ± 0.00
C24: 1 *n*-9 ^#^	—	0.02^a^ ± 0.00	0.02^a^ ± 0.00	0.01^b^ ± 0.00
C18: 2 *n*-6 (LA)^&^	0.07^d^ ± 0.00	0.45^b^ ± 0.01	0.51^a^ ± 0.00	0.38^c^ ± 0.01
C20: 2 *n*-6^&^	0.01^b^ ± 0.00	0.01^b^ ± 0.00	0.02^a^ ± 0.00	0.02^a^ ± 0.00
C18: 3 *n*-3 (ALA)^&^	0.05^a^ ± 0.00	—	—	0.04^b^ ± 0.00
C20: 3 *n*-3^&^	0.01^c^ ± 0.00	0.02^b^ ± 0.00	0.03^a^ ± 0.00	0.03^a^ ± 0.00
C20: 3 *n*-6^&^	0.02^a^ ± 0.00	—	0.01^c^ ± 0.00	0.01^b^ ± 0.00
C20: 4 *n*-3 (AA)^&^	0.04 ± 0.00	—	—	—
C20: 5 *n*-3 (EPA)^&^	0.02^d^ ± 0.00	0.11^b^ ± 0.00	0.13^a^ ± 0.00	0.07^c^ ± 0.00
C22: 6 *n*-3 (DHA)^&^	0.04^d^ ± 0.00	0.47^c^ ± 0.01	0.72^a^ ± 0.02	0.57^b^ ± 0.01
SFA	1.06^c^ ± 0.00	3.60^a^ ± 0.03	3.45^a^ ± 0.01	2.57^b^ ± 0.03
MUFA	0.97^c^ ± 0.01	4.23^ab^ ± 0.03	4.83^a^ ± 0.01	3.61^b^ ± 0.05
PUFA	0.26^c^ ± 0.00	1.06^b^ ± 0.00	1.42^a^ ± 0.02	1.11^b^ ± 0.02
TUFA	1.22^c^ ± 0.00	5.29^ab^ ± 0.03	6.25^a^ ± 0.02	4.72^b^ ± 0.03
PUFA/SFA	0.25^b^ ± 0.00	0.29^b^ ± 0.00	0.41^a^ ± 0.01	0.43^a^ ± 0.01
EPA + DHA	0.06^d^ ± 0.00	0.58^c^ ± 0.01	0.85^a^ ± 0.02	0.64^b^ ± 0.01
∑*n*-3	0.16^d^ ± 0.00	0.60^c^ ± 0.01	0.88^a^ ± 0.02	0.71^b^ ± 0.02
∑*n*-6	0.10^c^ ± 0.00	0.46^b^ ± 0.01	0.54^a^ ± 0.01	0.41^b^ ± 0.01
*n*-3/*n*-6	1.59^b^ ± 0.01	1.30^c^ ± 0.02	1.64^ab^ ± 0.03	1.73^a^ ± 0.03
DHA/EPA	2.0^d^ ± 0.01	4.27^c^ ± 0.02	5.54^b^ ± 0.03	8.14^a^ ± 0.03

Note: C12: 0, lauric acid; C14: 0, myristic acid; C15: 0, pentadecanoic acid; C16: 0, palmitic acid; C17: 0, heptadecanoic acid; C18: 0, stearic acid; C21: 0, heneicosanoic acid; C24: 0, lignoceric acid; C14: 1 *n*-5, myristoleic acid; C16: 1 *n*-7, palmitoleic acid; C17: 1 *n*-7, heptadecanoic acid; C18: 1 *n*-9, oleic acid; C20: 1 *n*-9, eicosenoic acid; C24: 1 *n*-9, nervonic acid; C18: 2 *n*-6, linoleic acid; C20: 2 *n*-6, eicosadienoic acid; C18: 3 *n*-3, α-linolenic acid; C20: 3 *n*-3, 11, 14, 17-eicosatrienoic acid; C20: 3 *n*-6, 8, 11, 14-eicosatrienoic acids; C20: 4 *n*-3, arachidonic acid; C20: 5 *n*-3, eicosapentaenoic acid, EPA; C22: 6 *n*-3, docosahexaenoic acid, DHA; SFA, saturated fatty acids; MUFA, monounsaturated fatty acids; PUFA, polyunsaturated fatty acids; TUFA, total unsaturated fat acids.

The different letters (a, b, c, and d) in the same line indicated significant differences (*p* < 0.05).

^*^For saturated fatty acids.

^#^For monounsaturated fatty acids.

^&^For polyunsaturated fatty acids.

—represents not detected.

A growing number of studies have shown that DHA plays an important role in normal retina and brain development [25]. Although LA can be converted into EPA in the human body, the rate of this reaction in the human body is very slow and the amount of conversion is very small, far from meeting the human body’s needs [35]. Therefore, it must be directly supplemented from food. Fish is a good food source for DHA and EPA. The American Heart Association (AHA) suggests that people who are diagnosed with coronary heart disease (CHD) should intake approximately 1 g of DHA and EPA every day. People without CVD should intake approximately 500 mg of these acids each day for prophylactic purposes. Higher doses of DHA and EPA were reported to decrease high triglyceride levels in the blood [36]. The AHA suggests that a daily intake of approximately 2–4 g of these acids can lower triglycerides. Whelen [37] reported that high ratio of DHA/EPA has an advantageous impact on consumer health and that DHA is more efficient than is EPA in reducing the risk of CHD. The contents of EPA and DHA in the cultivated spiny loaches were significantly higher than that of the wild (0.06 ± 0.00 g/100 g fresh weight), and the second age of cultivation demonstrated the highest content (0.85 ± 0.02 g/100 g fresh weight). Higher ratio of DHA/EPA was measured in the cultivated spiny loaches, especially in the second/third stage (5.54 or 8.14). Above results suggested that cultivated spiny loaches, especially at the second age was the good source of EPA and DHA.

### 3.3 Amino acid compositions

As shown in Table 3, a total of 16 amino acids were detected in the wild and cultivated spiny loach, including seven EAA and nine non-EAA. The result was similar to those reported from fish of *Sinogastromyzon szechuanensis* (wild, Neijiang section of Sichuan, China) [38] and *Triplophysa dalaica* (wild, Zijiang of Hunan, China) [39]. The type and content of amino acids can reflect the quality of food protein, and the content of total EAA (TEAA) is the most important index to evaluate the nutritional value [40]. In the present study, the TAA and TEAA contents of the second-age and the wild spiny loaches are not significantly different, and both were significantly higher than the first and third age, showing that the cultivated second-year-old spiny loaches had the best nutritional value. The results suggested that spiny loaches at different breeding stages demonstrated great difference in amino acids, which might be due to that the demand for protein in feed is different in different cultivation stages, and the current feed was more conducive to the growth and development of spiny loach at the second age. Previously, the amino acid content of cultured fish was lower than that of the wild one [41,42], in recent years, with the improved breeding technology, good water and feed quality, the nutritional value of cultivated spiny loaches at suitable stage has been improved.

**Table 3:** Amino acid compositions between the wild and cultivated spiny loaches (g/100 g fresh weight)

Amino acids	Wild spiny loaches	Cultivated spiny loaches (1st year)	Cultivated spiny loaches (2nd year)	Cultivated spiny loaches (3rd year)
Lys*	1.44^a^ ± 0.03	1.38^b^ ± 0.01	1.48^a^ ± 0.04	0.79^c^ ± 0.01
Thr*	0.73^a^ ± 0.01	0.69b ± 0.01	0.74^a^ ± 0.01	0.39^c^ ± 0.01
Val*	0.74^b^ ± 0.02	0.72b ± 0.01	0.78^a^ ± 0.01	0.43^c^ ± 0.01
Met*	0.38^a^ ± 0.03	0.32^b^ ± 0.00	0.40^a^ ± 0.01	0.18^c^ ± 0.03
Ile*	0.73^ab^ ± 0.02	0.71^b^ ± 0.01	0.74^a^ ± 0.02	0.39^c^ ± 0.01
Leu*	1.24^a^ ± 0.02	1.19^b^ ± 0.01	1.26^a^ ± 0.02	0.65^c^ ± 0.01
Phe*	0.63^b^ ± 0.01	0.63^b^ ± 0.00	0.65^a^ ± 0.02	0.35^c^ ± 0.01
Glu^&#^	2.92^a^ ± 0.03	2.60^c^ ± 0.01	2.78^b^ ± 0.06	1.41^d^ ± 0.03
Asp^&#^	1.63^a^ ± 0.07	1.64^a^ ± 0.02	1.66^a^ ± 0.02	0.85^b^ ± 0.00
Ala^&#^	1.08^a^ ± 0.01	1.03^b^ ± 0.01	1.04^b^ ± 0.02	0.54^c^ ± 0.01
Gly^&#^	0.87^a^ ± 0.06	0.82^a^ ± 0.01	0.76^b^ ± 0.02	0.40^c^ ± 0.01
Tyr^&^	0.40^b^ ± 0.01	0.37^c^ ± 0.01	0.42^a^ ± 0.00	0.22^d^ ± 0.01
Ser^&^	0.67^a^ ± 0.00	0.63^b^ ± 0.01	0.67^a^ ± 0.01	0.35^c^ ± 0.01
Pro^&^	0.55^a^ ± 0.02	0.51^b^ ± 0.00	0.44^c^ ± 0.01	0.25^d^ ± 0.02
His^&^	0.46^b^ ± 0.00	0.51^a^ ± 0.01	0.50^a^ ± 0.01	0.28^c^ ± 0.01
Arg^&^	0.95^a^ ± 0.01	0.85^c^ ± 0.01	0.91^b^ ± 0.00	0.45^d^ ± 0.01
TAA	15.42^a^ ± 0.14	14.58^b^ ± 0.09	15.23^a^ ± 0.15	7.93^c^ ± 0.00
TEAA	5.90a ± 0.12	5.63b ± 0.03	6.05^a^ ± 0.10	3.18^c^ ± 0.01
TNEAA	9.52^a^ ± 0.02	8.95^c^ ± 0.05	9.18^b^ ± 0.05	4.75^d^ ± 0.01
TFAA	6.50^a^ ± 0.04	6.09^c^ ± 0.04	6.24^b^ ± 0.04	3.20^d^ ± 0.01
TEAA/TAA	0.39	0.39	0.40	0.41
TEAA/TNEAA	0.62	0.63	0.66	0.67
TFAA/TAA	0.42	0.42	0.41	0.40

Note: TAA, total amino acids; TEAA, total essential amino acids; TNEAA, total nonessential amino acids; TFAA, total flavor amino acids.

The different letters (a, b, c and d) in the same line indicated significant differences (*p* < 0.05).

^*^Eessential amino acids.

^&^Non-essential amino acids.

^#^Delicious amino acids.

Flavor amino acids include Glu, Asp, Ala and Gly, which determine the flavor taste of the food protein. Glu and Asp are umami amino acids, while Gly and Ala are sweet amino acids. As shown in Table 3, the ratios of them to TAA in the wild spiny loaches were 0.42, 0.42, 0.41 and 0.40, respectively, and the contents of Glu and Asp were significantly higher than Gly and Ala. This is the reason that the loach tastes delicious. The contents of umami amino acids in the wild and second-age spiny loaches were higher, 6.50 ± 0.04 and 6.24 ± 0.04 g/100 g fresh weight, respectively, and the lowest in the third age group was 3.20 ± 0.01 g/100 g fresh weight. It is worth mentioning that the contents of TEAA and flavor amino acids of the second-age spiny loaches were significantly higher than that of the other two stages. As the fodder in different cultivated stages of spiny loach was the same, the highest content detected in the second-age spiny loaches might be due to the higher absorption and conversion efficiency of protein.

### 3.4 Protein and nutritional quality evaluation

According to the FAO/WHO amino acid pattern, the TEAA/TAA value is about 0.4, and the TEAA/TNEAA value should exceed 0.6. In this study, the TEAA/TAA values of wild and three cultivated-age spiny loaches were 0.39, 0.39, 0.40 and 0.41, respectively, and the TEAA/TNEAA values were 0.62, 0.63, 0.66 and 0.67. These results suggested that the wild and all three cultivated spiny loaches met the FAO/WHO amino acid pattern standard and belong to the high-quality protein source food, especially for the cultivated spiny loaches in the second and third age. As shown in Table 4, the TEAA content only in the second-age group (2201.28) was higher than that at FAO/WHO amino acid pattern (2190), and the third-age group was the lowest (1132.13). According to AAS and CS scores, Lys content in the first (454.67) and second-age spiny loaches (503.54) far exceeded the requirement of FAO/WHO amino acid pattern (340) and whole egg amino acid pattern (441). Besides, Thr (251.77) and Ile (251.77) only in the second-age group were higher than the FAO/WHO amino acid pattern (250). The first limiting amino acid was Met, both in the wild and cultivated spiny loaches. Notably, the EAAI value in the cultivated second-age group was also the highest among the four groups. Higher EAAI indicates more reasonable amino acid composition, better protein quality and higher utilization [43]. All these results suggested that the protein quality of the second-age loach was better than that of the other three groups. The mixtures of branched-chain and aromatic amino acids have the liver protection effect, and the *F* value (molar ratios of branched-chain amino acids to aromatic amino acids) of normal people is 3. 0–3. 5, when the liver is damaged, it is reduced to 1. 0–1. 5. In this study, the *F* values of the cultivated loaches at three age stages were 2.62, 2.60 and 2.58, which were higher than those of the wild spiny loach (1.43). To sum up, the amino acid content and ratios of the second-age stage were more responsive for human needs to prevent against diseases.

**Table 4:** Evaluation of amino acid value of the wild and cultivated spiny loaches

	F/W standard (mg/g)	WEP standard (mg/g)	Wild spiny loaches (mg/g)			Cultivated spiny loaches (1st year) (mg/g)			Cultivated spiny loaches (2nd year) (mg/g)			Cultivated spiny loaches (3rd year) (mg/g)		
			AAC	AAS	CS	AAC	AAS	CS	AAC	AAS	CS	AAC	AAS	CS
Thr	250	292	240.51	0.96	0.82	221.49	0.89	0.76	251.77	1.01	0.86	129.86	0.52	0.44
Val	310	410	243.81	0.79	0.59	231.12	0.75	0.56	265.38	0.86	0.65	143.18	0.46	0.35
Leu	440	534	408.54	0.93	0.77	382.00	0.87	0.72	428.69	0.97	0.80	216.44	0.49	0.41
Ile	250	331	240.51	0.96	0.73	227.91	0.91	0.69	251.77	1.01	0.76	129.86	0.52	0.39
Lys	340	441	474.43	1.40	1.08	454.67	1.34	1.03	503.54	1.48	1.14	263.05	0.77	0.60
Met	220	386	125.20	0.57	0.32	102.72	0.47	0.27	136.09	0.62	0.35	59.94	0.27	0.16
Phe＋Tyr	380	565	339.35	0.89	0.60	321.01	0.84	0.57	364.04	0.96	0.64	189.80	0.50	0.34
Total	2,190	2,959	2072.35			1940.93			2201.28			1132.13		
EAAI			67.55			62.18			71.82			36.30		
*F*			1.43			2.62			2.60			2.58		

Note: AAC, amino acids content; F/W standard, FAO/WHO standard; WEP standard, whole egg protein standard; EAAI, essential amino acid index; AAS, amino acid score; CS, chemical score.

### 3.5 Volatile compounds

There are also numerous studies showing that volatile compounds play a significant role in the quality of fish products and are key factors in consumers’ acceptance [44,45]. Therefore, the volatile component analysis of spiny loaches is of great significance for nutritional value assessment and safety management.

As presented in Table 5, 11 volatile compounds were detected in the wild spiny loach, while 13 were detected in the farmed spiny loaches. The detected volatile flavor compounds were mainly alkanes, the others were alcohols, esters and aldehydes. Alanane compounds had fragrant and sweet flavors. The higher content in alkanes was *N*-decane, which was 13.93 ± 0.92% in the wild spiny loaches, and was found to be the highest in the second-age cultivated spiny loaches (22.39 ± 0.93%). *N*-butyl cyclopentane, dimethylbenzene, dibutyl hydroxytoluene, 1-methylcyclohexanol and propyl caproate were detected only in the cultivated spiny loach, while 1,4-dibutylbenzene and butyrate were present only in the wild. This might be due to the difference in forage between cultivated and wild ones. Most of the saturated C_6_–C_12_ aldehyde compounds have the fragrance of grass and fat, with a low threshold and a strong correlation with fish flavor [46,47]. As can be seen from the results presented in Table 5, octanal (C_8_H_18_O) was detected in all four groups of fish, and was the most abundant in the second-age group (7.11 ± 0.27%).

**Table 5:** Composition of volatile compounds (% of area) between wild and cultivated spiny loaches

Volatile compounds	Wild spiny loaches	Cultivated spiny loaches (1st year)	Cultivated spiny loaches (2nd year)	Cultivated spiny loaches (3rd year)
*N*-Butyl cyclopentane	—	9.93%^b^ ± 0.74%	2.62%^c^ ± 0.23%	14.64%^a^ ± 0.48%
*N*-Decane	13.93%^d^ ± 0.92%	20.7%^b^ ± 1.72%	22.39%^a^ ± 0.93%	19.02%^c^ ± 0.46%
Dimethylbenzene	—	2.58%^a^ ± 0.39%	2.06%^b^ ± 0.10%	1.93%^b^ ± 0.12%
Laurane	6.13%^a^ ± 0.15%	2.61%^b^ ± 0.20%	6.15%^a^ ± 0.49%	2.94%^b^ ± 0.03%
1,2,4,5-Tetratoluene	3.41%^a^ ± 0.11%	2.30%^c^ ± 0.04%	2.66%^b^ ± 0.04%	2.09%^d^ ± 0.05%
Naphthalene	13.65%^a^ ± 0.15%	8.16%^c^ ± 0.08%	9.64%^b^ ± 0.07%	9.37%^b^ ± 0.34%
1-Methylnaphthalene	34.41%^a^ ± 1.68%	17.37%^b^ ± 1.67%	18.28%^b^ ± 0.16%	17.74%^b^ ± 0.58%
Dibutyl hydroxytoluene	—	21.09%^a^ ± 1.50%	17.30%^b^ ± 0.22%	17.66%^b^ ± 0.72%
1,4-Dibutylbenzene	3.37% ± 0.04%	—	—	—
1-Methylcyclohexanol	—	2.75%^b^ ± 0.57%	3.21%^a^ ± 0.21%	2.09%^c^ ± 0.16%
1-Nonene-4-ol	6.69%^a^ ± 0.10%	3.43%^b^ ± 0.07%	3.07%^c^ ± 0.01%	3.68%^b^ ± 0.06%
Menthol	6.00%^a^ ± 0.27%	2.35%^bc^ ± 0.17%	2.65%^b^ ± 0.05%	2.14%^c^ ± 0.15%
2-Methylbutanol	2.54% ± 0.30%	—	—	—
Propyl caproate	—	2.65%^b^ ± 0.39%	2.86%^ab^ ± 0.13%	3.18%^a^ ± 0.24%
Butyrate	5.42% ± 0.41%	—	—	—
Octanal	4.45%^b^ ± 0.46%	4.08%^bc^ ± 0.42%	7.11%^a^ ± 0.27%	3.52%^c^ ± 0.10%

Note: The different letters (a, b, c and d) in the same line indicated significant differences (*p* < 0.05).

—represents not detected.

## 4 Conclusions

In general, this study provides a detailed analysis and comparison of the nutritional composition between wild and cultivated spiny loaches at three different cultivation stages, including proximate components, fatty acids, amino acids and volatile flavor compounds. The cultivated spiny loaches had higher energy and fat contents, especially for the second-age one. The total contents of polyunsaturated fatty acids and EPA + DHA in the second-age spiny loaches were the highest, as well as the essential and flavor (Glu, Asp, Ala and Gly) amino acids, and EAAI value. Similarly, the types and contents of volatile substances in the second-age spiny loaches were relatively high. Therefore, the nutritional value of cultivated spiny loaches at the second breeding age is higher than that of the wild one. This study would provide a theoretical basis for consumers to choose right-age cultivated loaches, and appropriate cultivation period for farmers to improve the quality of cultivated loaches from the perspective of nutritional value.
